# Left Ventricular Outflow Tract Tachycardias

**DOI:** 10.1016/s0972-6292(16)30583-6

**Published:** 2013-01-01

**Authors:** Johnson Francis

**Affiliations:** Senior Consultant Interventional Cardiologist, Baby Memorial Hospital, Calicut, Kerala, India

**Keywords:** Left Ventricular Outflow Tract Tachycardias, Radiofrequency Catheter Ablation

Left ventricular outflow tract tachycardia (LVOT-VT) is an uncommon type of idiopathic left ventricular tachycardia (ILVT) thought to be due to cyclic adenosine mono phosphate (c-AMP) mediated triggered activity. The arrhythmia can be terminated pharmacologically with calcium channel blockers or beta-blockers [1]. LVOT-VT can arise above or below the coronary cusps and the former variety can also be called as aortic cusp VT. An S wave in lead I and a precordial R wave transition at V1 or V2 is common to both varieties of LVOT-VT. Absence of S wave in V5 or V6 may suggest supra cuspal origin while an Rs pattern in V5 or V6 may indicate an infracuspal origin [2].

Yet another classification of LVOT-VT [3] was into VT originating from aortic cusps, from aortomitral continuity, anterior site around the mitral annulus and the epicardium. In case of aortic cusps, VT usually arises only form the right and left coronary cusps due to the extension of ventricular muscle fibers into their bases, while it seldom arises from the non-coronary cusp, which lacks these fibers and is composed of fibrous tissue.

Tachycardiomyopathy, though much more common with supraventricular arrhythmias, has also been described with ventricular arrhythmias. Most of these reports were in case of right ventricular out flow tract tachycardias (RVOT-VT). In this issue of the journal, Mora G et al [4] describe the reversal of tachycardiomyopathy after successful ablation of LVOT-VT. In their patient, left ventricular ejection fraction improved from 35% before ablation to 70% at one month after ablation. There was also a reversal of left ventricular dilatation, with absence of symptoms.

Various approaches have been described for the ablation of LVOT-VT. Shimoike E, et al [5] mapped the origin of two cases to the posterior LVOT corresponding to the aortomitral continuity or left fibrous trigone and successfully ablated them. Successful termination of the tachycardia was obtained by basal septal ablation in one case by Chiladakis JA et al [6]. Trans coronary cusp RF ablation has been achieved in case of LVOT-VT arising near the left coronary sinus [7]. A minimally invasive surgical approach may be useful in LVOT-VT with an epicardial origin when an endocardial approach fails [8]. Such VTs can be mapped through the anterior interventricular vein and ablation through the vein can also be successful [9]. Some of the VTs originating near the anterior epicardial veins can also be ablated form the left coronary sinus or nearby LV endocardium [10].

Advanced technologies like intracardiac echocardiography [11], three dimensional non contact mapping [12] and magnetic electroanatomic mapping [1,13] have all been used in the successful mapping and ablation of LVOT-VT. Ablation of LVOT-VT may not be always successful and free of complications. Campos B et al had 56% success rate in their 16 cases of VT originating from the left sinus of Valsalva near the anterior epicardial veins [10]. Turkoglu C and colleagues [14] reported chronic total occlusion of left circumflex coronary artery after RF ablation of LVOT-VT.
